# T Cells as an Emerging Target for Chronic Pain Therapy

**DOI:** 10.3389/fnmol.2019.00216

**Published:** 2019-09-11

**Authors:** Geoffroy Laumet, Jiacheng Ma, Alfred J. Robison, Susmita Kumari, Cobi J. Heijnen, Annemieke Kavelaars

**Affiliations:** ^1^Department of Physiology, Michigan State University, East Lansing, MI, United States; ^2^Laboratories of Neuroimmunology, Department of Symptom Research, The University of Texas MD Anderson Cancer Center, Houston, TX, United States

**Keywords:** chronic pain, T cells, cytokines, neuroimmune, opioids

## Abstract

The immune system is critically involved in the development and maintenance of chronic pain. However, T cells, one of the main regulators of the immune response, have only recently become a focus of investigations on chronic pain pathophysiology. Emerging clinical data suggest that patients with chronic pain have a different phenotypic profile of circulating T cells compared to controls. At the preclinical level, findings on the function of T cells are mixed and differ between nerve injury, chemotherapy, and inflammatory models of persistent pain. Depending on the type of injury, the subset of T cells and the sex of the animal, T cells may contribute to the onset and/or the resolution of pain, underlining T cells as a major player in the transition from acute to chronic pain. Specific T cell subsets release mediators such as cytokines and endogenous opioid peptides that can promote, suppress, or even resolve pain. Inhibiting the pain-promoting functions of T cells and/or enhancing the beneficial effects of pro-resolution T cells may offer new disease-modifying strategies for the treatment of chronic pain, a critical need in view of the current opioid crisis.

## Pain Modulation by Cytokines and Immune Cells

Pain is one of the cardinal signs of inflammation, and anti-inflammatory drugs are the first-line therapy in many acute and chronic pain conditions. In patients, chronic pain is often associated with signs of activation of the immune system as characterized by increased circulating levels of pro-inflammatory cytokines (Davies et al., 2007; Koch et al., 2007; Uçeyler et al., 2007a,b, 2011; Cameron and Cotter, 2008; Kraychete et al., 2010; Held et al., 2019). The circulating level of the anti-inflammatory cytokines interleukin (IL)-10 and IL-4 were higher in patients with painless neuropathy than in patients with painful neuropathy and controls (Uçeyler et al., 2007b; Held et al., 2019).

The immune system can be divided into two functional arms: the innate and adaptive immune systems. The contribution of the innate immune system (macrophages, neutrophils, microglia…) and proinflammatory cytokines to the transition from acute to chronic pain has been well established and reviewed elsewhere (Scholz and Woolf, 2007; Grace et al., 2014; McMahon et al., 2015; Ji et al., 2016; Chen et al., 2018; Baral et al., 2019). Innate immune cells and released cytokines modulate both peripheral and central sensitization, leading to pain hypersensitivity. Peripheral sensitization is defined as a reduction in the threshold of excitability of sensory neurons, which thus become hyperexcitable. One interesting property of some pro-inflammatory cytokines (e.g., IL-1β) is their ability to interact directly with pain-sensing neurons (nociceptors among sensory neurons) to sensitize them and render them hyperexcitable, increasing the afferent input into the spinal cord (Binshtok et al., 2008; Baral et al., 2019). Moreover, in the dorsal horn of the spinal cord, cytokines facilitate the development of central sensitization (enhanced responses of pain spinal circuits). For example, Tumor Necrosis Factor α (TNFα) enhances the frequency of spontaneous excitatory post-synaptic current in lamina II neurons of the spinal cord (Kawasaki et al., 2008). Central sensitization in the spinal cord is thought to contributes to the transition to chronic pain and the spreading of pain beyond the site of primary insult (Woolf and Salter, 2000; Ji et al., 2016).

The role of the adaptive immune cells is less clear. The adaptive immune system is comprised of B and T cells (lymphocytes), and a few recent findings point out a potential role for B cells in pain, mainly through the production of autoantibodies (Andoh and Kuraishi, 2004; Klein et al., 2012; Hunt et al., 2018). However, the present review focuses on the emerging role of T cells in pain.

## Overview of The T Cell Subsets

T cells express a unique antigen receptor complex on their surface: T cell receptor (TCR). In most T cells, the TCR is composed of two highly variable protein chains, α and β. The uniqueness of the TCR results from genetic rearrangements in the thymus driven by the proteins encoded by the recombination activating genes RAG1 and RAG2. The resulting unique TCRs have a very high degree of antigen specificity. TCR forms a complex with the co-receptor Cluster of Differentiation 3 (CD3) which is used as a marker to identify T cells. This TCR complex recognizes antigenic epitopes in the context of the Major Histocompatibility Complex (MHC). CD8+ T cells recognize antigen in the context of MHC-I that is expressed by virtually every nucleated cell, including neurons. In contrast, CD4+ T cells recognize MHC-II antigen which is presented specifically by antigen presenting cells (APC) such as macrophages, microglia, B cells and dendritic cells.

**Figure 1:**
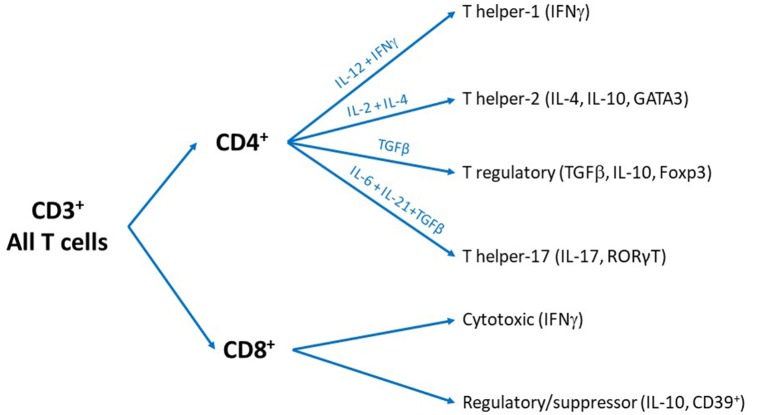
Overview of the different subsets of T cells. All T cells are cluster of differentiation 3 (CD3)+ and can be divided into two subsets: CD4+ and CD8+ T cells. We recognize that some T cells are CD3- or CD4- CD8- cells and other subsets exist but these particular phenotypes are beyond the scope of the present review.

The CD4+ T cells are so-called T helper (Th) cells because they help cells from both the innate and adaptive immune system to optimize their response. CD4+ T cells can differentiate into functionally different subsets including Th1, Th2, Th17 or regulatory T cells (Treg; Zhu and Paul, 2008). These subsets differ from each other in their pattern of cytokine production and specific expression of characteristic transcription factors. Briefly, Th1 cells express T-bet and signal transducer and activator of transcription (STAT) 4 and release gamma-Interferon (IFNγ) and IL-2; Th2 cells express GATA3 and STAT5 and release IL-4, IL-10 and IL-13; Th17 cells express RORγT and release IL-17; and Treg express forkhead box P3 (FOXP3) and release Tumor Growth Factor (TGF)β and IL-10. Treg are a very interesting subset of T cells as their main role is to suppress the activity of other immune cells including the other subsets of T cells. The cytokines in the environment (Mousset et al., 2019), signaling through the antigen receptor, and level of engagement of specific co-stimulatory and co-inhibitory molecules on the cell surface of T cells orientate the fate of activated CD4+ T cells to specific helper subset. For example, high concentrations of IL-12 + IFNγ instruct the naïve T cells to differentiate into a Th1 profile, while IL-4 + IL-2 promote Th2 and IL-6 + IL-21 + TGFβ instruct toward Th17 subset differentiation. The anti-inflammatory cytokine TGFβ turns the cells toward the Treg fate (Figure 1). Other subsets of CD4+ T cells have been identified such as Th9, Th22, follicular T cell and Natural killer T cell (NKT; Zhu and Paul, 2008; Hirahara and Nakayama, 2016; Mousset et al., 2019), but will not be discussed in this review because their contribution to pain is completely unknown.

The CD8+ T cells can be differentiated into cytotoxic T cells (CTL) and suppressor/regulatory T cells. The best characterized role of the CD8+ T cells is to kill virus-infected and tumor cells. The CTLs carry out all the attention of research on the CD8+ T cells, but the suppressor/regulatory CD8+ T cells have often been neglected. The role of the different phenotypes of CD8+ T cells (Tc1, Tc2, Tc9, Tc17) and memory status (effector, central memory, effector memory…; Mousset et al., 2019) has not been investigated in chronic pain and thus will not be discussed in the present review.

Another type of T cell is the γδ T cells, which have a distinct TCR. In contrast to the αβ-TCR, γδ-TCR are invariant and less abundant (Itohara et al., 1993). In the circulation, only 5% of T cells are γδ T cells (Glusman et al., 2001), but they represent a high proportion of gut- and skin-resident immune cells, where they are localized near the sensory neurons (Marshall et al., 2019).

## Phenotype of Circulating T Cells in Patients With Chronic Pain

Few studies have analyzed circulating T cell counts and subsets in patients with chronic pain. Those studies are often small, and the parameters analyzed vary. Studies in patients with chronic pain do not report changes in the total number of circulating T cells compared to pain-free matched controls (Mangiacavalli et al., 2010; Luchting et al., 2015). Likewise, the number of circulating CD4+ and CD8+ T cells seems unchanged in various chronic pain conditions (Brennan et al., 1994). However, in patients with chronic headache, a lower number of CD8+ T cells, and consequently higher CD4+/CD8+ ratio was found compared to control individuals (Gilman-Sachs et al., 1989).

In general, assessment of the total number of circulating T cell subsets in patients with chronic pain is not very informative. To gain more insight into the role of T cells in chronic pain, some studies investigated the functional subsets of CD4+ T cells. These studies found an imbalance in the ratio of Th1/Th2 (Liu et al., 2006; Mangiacavalli et al., 2010) and Th17/Treg ratio (Tang et al., 2013; Luchting et al., 2014, 2015). To avoid bias, the absence of infection was controlled in these patients. Contrary to the expected pro-inflammatory profile, these studies actually found indication of an anti-inflammatory shift in T cell profile toward Th2 and Treg. Consistently, the expression of the specific Th17 transcription factor RORγT and cytokine IL-17 were decreased as well in complex regional pain syndrome (CRPS) patients (Haas et al., 2011; Heyn et al., 2019). In another study in CRPS patients, the number of Tregs did not change, but the specific sub-subset of CD39+ Treg was decreased (Heyn et al., 2019). In contrast, a stronger Th1 response was observed in T cells from patients with neuropathic pain as compared to controls when the cells were stimulated *in vitro* with myelin-derived antigen (Diederich et al., 2018). Further analysis reported changes in specific markers for sub-subsets of T cells. Furthermore, smoking affects both the development of chronic pain and T cell phenotypes (Scott et al., 1999; Power et al., 2001; Vargas-Rojas et al., 2011), strengthening the argument for a connection. In patients with chronic pain, smoking increased the Th17/Treg ratio measured by flow cytometry and mRNA expression of RORγT and FOXP3, and this increased Th17/Treg ratio was associated with higher pain sensitivity (Heyn et al., 2018).

Given that T cells are easy to access peripherally, they represent an attractive pool for identification of potential biomarkers to survey the development of chronic pain. However, the clinical relevance of measuring circulating T cells is not yet clear, and additional studies are necessary to identify potential biomarkers. It is also important to note that the phenotype of T cells can be affected by pain-killers (e.g., morphine; Ranganathan et al., 2009; Wiese et al., 2016; Plein and Rittner, 2018), potentially complicating any findings in patients after they begin treatment.

## T Cells in Neuroimmune Interactions

T cells play an important role in the communication between the nervous and immune systems, and one of the most studied interactions between T cells and the nervous system is the anti-inflammatory reflex (Tracey, 2009). During systemic inflammation, proinflammatory cytokines activate the afferent vagus nerve which initiates a reflex response. β2-adrenergic receptor-expressing T cells react to noradrenaline released by the sympathetic splenic nerve, triggering the production of acetylcholine by T cells. Acetylcholine signals to macrophages to switch from the production of pro-inflammatory to anti-inflammatory cytokines such as IL-10, thus dampening the immune response (Pavlov and Tracey, 2017). The anti-inflammatory reflex is absent in nude mice lacking T cells, and adoptive transfer of T cells restores the anti-inflammatory reflex, confirming the crucial role of T cells in this neuroimmune communication (Rosas-Ballina et al., 2011).

T cell function is also directly influenced by nociceptors. Upon activation, nociceptors release glutamate, calcitonin gene-related peptide (CGRP), and Substance P (SP). The canonical role of theses neurotransmitters and neuropeptides is to activate second order neurons in the dorsal horn of the spinal cord to signal pain into the central nervous system (CNS). In addition to this neuronal transmission role, activated nociceptors release these neurotransmitters and neuropeptides at their peripheral endings, regulating activity of local immune cells including T cells. T cells express inotropic and metabotropic glutamate receptors, SP and CGRP receptors (Rameshwar et al., 1992; Ganor et al., 2003; Mikami et al., 2011; Ohtake et al., 2015; Szklany et al., 2016). Activation of these receptors regulates various T cell functions such as adhesion, chemotactic migration, proliferation and immunological phenotypes (Hosoi et al., 1993; Levite et al., 1998; Hood et al., 2000; Levite, 2000; Talme et al., 2008; Mikami et al., 2011). Not surprisingly, nociceptor–T cell interaction has a critical role in chronic inflammatory diseases and in immune defense against infection (Basbaum and Levine, 1991; Razavi et al., 2006; Chiu et al., 2013; Cohen et al., 2019). Genetic ablation of nociceptors alters the immune response to sterile injury or infection and pathogen control (Chiu et al., 2013; Talbot et al., 2015; Baral et al., 2019).

Critically, the interaction between T cells and the nervous system is bidirectional, and T cells regulate neuronal function in the central and peripheral nervous systems. For instance, meningeal T cells secrete IL-4 to trigger brain derived neurotrophic factor (BDNF) production to enhance neurogenesis in the brain (Ziv et al., 2006). In an inflammatory skin disease model, Th2 cells trigger itch by secretion of IL-31, which binds to its receptor on sensory neurons, triggering calcium release, phosphorylation of ERK1/2 and activation of TRPA1 channel, driving neuronal activation and itch (Cevikbas et al., 2014).

Given the role of T cells in neuroimmune interactions, they likely have an important impact on the transition from acute to chronic pain. To identify the role of T cells in chronic pain, multiple pain models have been used, including models of nerve injury-induced neuropathic pain, inflammatory pain, and chemotherapy-induced peripheral neuropathy (CIPN). In this review, we will not discuss data collected from models of autoimmune disorders, such as multiple sclerosis, because the key role of T cells in autoimmunity itself makes it difficult to disentangle the specific role of T cells in pain in these models (Dendrou et al., 2015).

## Contribution of T Cells to Pain Sensitivity in Naïve Animals

**Figure 2:**
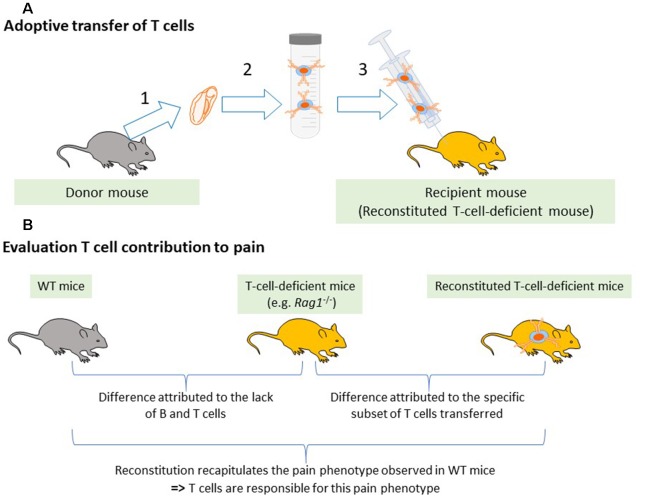
Reconstitution of T-cell-deficient mice. **(A)** Adaptive transfer of T cells from WT mice to T cell-deficient mice. (1) Lymphoid tissues such as the spleen, lymph nodes, or blood are collected from donor mice. (2) T cells or specific subsets of T cells are isolated using magnetic beads. (3) Selected T cells are injected into T-cell-deficient mice. **(B)** Evaluation of T cell contribution to pain. To attribute a pain phenotype to a function of T cells, the pain behavior in the immunodeficient mice must be different from the WT mice and reconstitution of the immunodeficient mice with T cells must normalize the pain response.

The contribution of T cells to pain can be evaluated by comparing pain-related behaviors in WT and T cell-deficient rodents. These animals often carry a genetic mutation in one of the genes involved in the rearrangement of the antigen receptor such as *Rag1*, *Rag2*, or Protein Kinase, DNA-Activated, Catalytic Subunit (*Prkdc* for severe combined immunodeficiency—SCID mice). Therefore, they lack the entire adaptive immune system (B and T cells). This lack of adaptive immune cells from birth may induce compensatory mechanisms and alter the innate immune cells and may even influence the neuronal circuitry (Filiano et al., 2016). On the other hand, the use of these transgenic animals is the cleanest way to deplete T cells preclinically. To critically evaluate the contribution of T cells, WT mice are compared to mice deficient for the whole adaptive immune system (simplified and referred to T-cell-deficient mice in this review) including the *Rag1*^−/−^, *Rag2*^−/−^, nude and SCID mice, as well as to mice reconstituted with specific populations of T cells (Figure 2).

At baseline, T-cell-deficient rodents are indistinguishable from control counterparts in response to mechanical stimuli in at least 3 different mouse genetic backgrounds (CD1, BALB and C57) and athymic rats (Moalem et al., 2004; Cao and DeLeo, 2008; Costigan et al., 2009; Vicuña et al., 2015; Krukowski et al., 2016; Rosen et al., 2017; Laumet et al., 2019). Reconstitution of T cell-deficient rodents with any type of T cells also does not alter the baseline pain sensitivity in male and female mice. Only one publication reported increased pain sensitivity in male and female mice that lack the adaptive immune system (Rosen et al., 2019), and an important difference is that this study included up to 40 animals per group while previous studies that did not observe differences investigated 5–10 mice per group. These findings suggest that there may be a small but statistically significant difference in baseline pain sensitivity between WT and T-cell-deficient mice.

## Contribution of T Cells to the Transition From Acute to Chronic Pain

The next paragraphs cover studies with male rodents unless otherwise indicated, and these data are summarized in Table 1 while the contribution to each CD4+ T cell subset is listed in Table 2. The potential role of sex differences is discussed at the end of this section. Advancing age is an important risk factor for chronic pain, and it is important to note that most of the studies discussed below were conducted in relatively young adult rodents.

**Table 1:** Pain hypersensitivity phenotypes in T-cell-deficient or T-cell-depleted rodents compared to WT or IgG-treated controls.

Chronic pain model	T cell depletion model	Sex	Pain sensitivity vs. controls	References
CCI	Nude (rats)	M	Reduced	Moalem et al. (2004)
CCI	*Rag1*^−/−^	M	Reduced	Kleinschnitz et al. (2006)
SNT	Nude and *Cd4*^−/−^	M	Reduced	Cao and DeLeo (2008)
SNI	*Rag1*^−/−^	M	Reduced	Costigan et al. (2009)
CCI	SCID	M	Identical	Labuz et al. (2010)
PSNL	Anti-CD4	M	Reduced	Kobayashi et al. (2015)
SNI	*Rag2*^−/−^	No info	Reduced	Vicuña et al. (2015)
SNI	*Rag1*^−/−^ and Nude	F	Identical	Rosen et al. (2017)
mSNI	Anti-αβTCR (rats)	M	Reduced	Du et al. (2018)
Paclitaxel	*Rag1*^−/−^	M	Prolonged	Krukowski et al. (2016)
Cisplatin	*Rag1*^−/−^ and *Rag2*^−/−^	M + F	Prolonged	Laumet et al. (2019)
CFA + OVA	Nude	No info	Prolonged	Boué et al. (2012)
CFA	TCRβ^−/−^	M	Identical	Ghasemlou et al. (2015)
CFA	*Rag1*^−/−^		Identical	Sorge et al. (2015)
CFA	*Rag2*^−/−^	M	Prolonged	Basso et al. (2016)
CFA	*Rag1*^−/−^ and Nude	F	Identical	Rosen et al. (2017)
CFA	TCRδ^−/−^	M + F	Identical	Petrović et al. (2019)
CFA	*Rag2*^−/−^	M	Prolonged	Laumet et al. (2016)
DSS visceral pain	SCID	No info	increased	Boué et al. (2014)
Formalin	*Tcrd*^−/−^	M + F	Identical	Petrović et al. (2019)
Formalin	Nude	M + F	increased	Rosen et al. (2019)
Morphine analgesia	*Rag1*^−/−^, *Cd4*^−/−^ and Nude	M + F	Reduced	Rosen et al. (2019)
Plantar incision	*Tcrb*^−/−^	M	Identical	Ghasemlou et al. (2015)
Plantar incision	*Tcrd*^−/−^	M + F	Identical	Petrović et al. (2019)

*CCI, chronic constriction injury; CFA, complete Freund’s adjuvant; DSS, dextran sulfate sodium; mSNI, modified SNI; OVA, ovalbumin immunization; PSNL, partial sciatic nerve ligation; SNI, spared nerve injury; SNT, spinal nerve transection; M, male; F, female*.

**Table 2:** Contribution of each CD4+ T cell subsets to pain sensitivity.

T helper subset	Pain sensitivity	Potential mechanisms	References
Th1	↑	Production of proinflammatory cytokines	Moalem et al. (2004) and Draleau et al. (2014)
Th2	↓	Production of anti-inflammatory cytokines (IL-10) and endogenous opioids	Moalem et al. (2004), Leger et al. (2011), Boué et al. (2014) and Basso et al. (2018)
Th17	↑	Production of proinflammatory cytokines and activation of microglia and macrophages	Kleinschnitz et al. (2006) and Huo et al. (2019)
Treg	↓	Production of anti-inflammatory cytokines (IL-10)	Austin et al. (2012), Liu et al. (2014), Lees et al. (2015) and Duffy et al. (2019)

### Nerve Injury-Induced Neuropathic Pain

In preclinical studies, neuropathic pain is usually induced by peripheral nerve injury through a surgical intervention (CCI, chronic constriction injury; SNI, spared nerve injury; SNL, spinal nerve injury; PSNL, partial sciatic nerve injury; SNT, spinal nerve transection).

#### Infiltration of T Cells Into the Nervous System Following Nerve Injury

Nerve injury generates an organized cascade of events to stimulate the inflammatory responses (Gadani et al., 2015). Immediately after injury, alarmins are released and glial cells surrounding the nerve are activated. In the following minutes, cytokines and chemokines are secreted, and neutrophils are recruited. Neutrophils are almost always the first peripheral immune cells to invade sites of injury. In hours to days, monocyte-derived macrophages will infiltrate the damaged nerve while T cells usually arrive days to weeks post-injury, first infiltrating the site of injury and distal part of the nerve, then the dorsal root ganglia (DRG, a cluster of the cell bodies of sensory neurons), and finally the dorsal horn of the spinal cord. Moalem et al. (2004) examined the kinetics of T cell infiltration of the sciatic nerve in response to CCI in rats. T cells were not observed in uninjured sciatic nerve (sham and contralateral nerve), and few T cells were detected at 3 days after injury. Significant T cell infiltration was observed at 7 days and peaked at 21 days at proximal (125 T cells/0.5 mm^2^ detected by anti-αβ TCR antibody) and distal sites of the injury (Moalem et al., 2004). Infiltrated T cells were still present at 40 days after injury (the last time point checked). This pattern is consistent with studies using different nerve injury models in rats and in mice, wherein few T cells were found at the site of injury at 3 days post-surgery and the number of T cells significantly increased from 7 to 28 days post-surgery (Cui et al., 2000; Kleinschnitz et al., 2006; Labuz et al., 2009; Austin et al., 2012; Kobayashi et al., 2015). T cells represented almost 10% of the infiltrating immune cells at 15 days after the injury (Labuz et al., 2009). Austin et al. (2012) reported 150–200 TCR+ cells/0.5 mm^2^ at the injury site at 28 days post-CCI.

Invading T cells may come from the circulation and are thought to penetrate the nerve from the endoneurial vasculature rather than migration across the nerve sheath (Eliav et al., 1999; Kobayashi et al., 2015). The infiltration of T cells appears to depend on phagocytic cells, as depletion of these cells using clodronate-liposome treatment prevented the infiltration of CD4+ T cells, suggesting that previous infiltration of innate immune cells is necessary for T cells to infiltrate the injured nerve.

Naïve DRGs lack a tight blood-nerve barrier and contain a low number of both CD4+ and CD8+ T cells (Austin et al., 2012; Liu et al., 2014; Vicuña et al., 2015; Krukowski et al., 2016). In contrast to the circulation, where 65%–70% of T cells are CD4+, in DRG 60%–70% are CD8+ T cells, indicating a regulated infiltration (McLachlan and Hu, 2014; Krukowski et al., 2016). The number of T cells increases in the DRG in response to both spinal and sciatic nerve injury (Hu and McLachlan, 2002; Austin et al., 2012; Du et al., 2018). Similar to the nerve, the number of T cells at 3 days post-surgery was not different between sham and injured DRGs but starts to increase after 7 days. The number of T cells increased 4–6 times at 28 days after nerve injury and persisted for at least 12 weeks (Hu and McLachlan, 2002). Interestingly, in this model, the T cells invading the DRG were mostly CD4+, inducing a switch in the CD4+/CD8+ ratio (McLachlan and Hu, 2014). The route taken by T cells to infiltrate into the DRG is still unknown. They possibly come from blood vessels or from the DRG and spinal meninges, specifically at the subarachnoid angle (Hu and McLachlan, 2002). Using IHC and lymphadenectomy approaches, a recent study demonstrated that after SNI, CD4+ T cells from lumbar lymph nodes begin migrating into the dorsal root leptomeninges to invade the DRG of the injured axons (Du et al., 2018). The lumbar sympathetic chain may be required for this migration (Hu and McLachlan, 2002; McLachlan and Hu, 2014), and lumbar DRG-invading T cells, mostly CD4+, are drained by the sciatic lymph node (McLachlan and Hu, 2014).

T cells are hardly detectable, if at all, in the spinal cords of naïve animals. However, as has been proposed for the brain, it is possible that T cells penetrate the CNS parenchyma but only in very small number and for a very short time, making them virtually undetectable (Kipnis et al., 2012). In response to injury, T cells may migrate into the spinal cord through the leptomeninges to reach the cerebral spinal fluid (CSF) as they infiltrate the dorsal root leptomeninges following nerve injury or in autoimmune disease (Schläger et al., 2016; Du et al., 2018). With immunostaining approaches, several studies observed the presence of CD4+ T cells in the dorsal horn of the spinal cord after PSNL, SNI and SNT (Cao and DeLeo, 2008; Costigan et al., 2009; Leger et al., 2011). However, even in these models, the number of T cells in the spinal cord stays very low. Flow cytometry experiments confirmed the presence of CD4+ T cells in the spinal cord at 7 days after nerve injury (Cao and DeLeo, 2008). In the SNT model, the phenotypes of infiltrated CD4+ cells are T-Bet+, IFNγ+, TNF-α+, and GM-CSF+, GATA3- or IL-4-, suggesting a Th1 phenotype (Draleau et al., 2014). The specific combination of adhesion molecules expressed in the spinal cord facilitates the infiltration of α4β1 integrin-expressing immune cells. Among T cells, Th1 cells have higher expression of α4β1, rendering them more likely to infiltrate the spinal cord than other T cell subsets (Rothhammer et al., 2011). In contrast, another study using staining with anti-CD2 to label all T cells and anti-CD8 to identify this specific subset did not observe T cell infiltration in the dorsal horn spinal cord from day 2 to 42 post-SNI (Gattlen et al., 2016). Thus, there are conflicting reports as to whether and how subsets of T cells enter the spinal cord in response to pain or injury, and further studies in this area will be critical.

In healthy conditions, T cells are virtually absent of the brain parenchyma but are present in the surrounding meninges (Kipnis et al., 2012). To our knowledge, the potential infiltration of T cells into brain areas associated with pain has not been investigated.

#### Contribution of T Cells to Nerve-Injury Induced Pain Hypersensitivity

The contribution of T cells to chronic pain can be investigated in WT mice by depletion or neutralization of T cells with antibodies. Administration of anti-CD4 antibody to deplete mice of functional CD4+ T cells, starting 4 days before surgery, reduced pain sensitivity following PSNL (Kobayashi et al., 2015). Repetitive intrathecal injections of anti-αβ-TCR antibody to deplete mice of all functional αβ T cells starting at 3 days post-SNI alleviated mechanical pain hypersensitivity as well. Interestingly, mechanical allodynia returned once the treatment was terminated and T cells may have repopulated the mouse (Du et al., 2018). One of the pioneer studies to use T cell-deficient animals (athymic rats) investigated the contribution of T cells to neuropathic pain induced by CCI. Nude rats developed reduced thermal and mechanical pain hypersensitivity compared to WT following CCI (Moalem et al., 2004). Reconstitution of athymic nude rats with IFNγ and IL-2 producing Th1 cells restored the pain behavior, while reconstitution with Th2 cells producing the anti-inflammatory cytokines IL-10, IL-4 and IL-13 further reduced thermal pain sensitivity after CCI (Moalem et al., 2004). In mice, a first investigation found that Rag1^−/−^ mice developed similar mechanical pain but reduced thermal pain hypersensitivity after CCI compared to WT mice (Kleinschnitz et al., 2006). Another study reported that mechanical allodynia was completely prevented in *Rag1*^−/−^ mice following SNI (Costigan et al., 2009). Reconstitution of T-cell-deficient mice with T cells (as done with athymic rats previously) is a necessary experiment to attribute the observed effects to the lack of T cells, as *Rag1*^−/−^ mice also lack B cells. This issue has been addressed by Cao and Deleo, as they observed that nude mice have reduced pain sensitivity after SNT and reconstitution of nude mice with CD4+ T cells restored pain hypersensitivity (Cao and DeLeo, 2008). Further, the aggravating effect of T cells on neuropathic pain was confirmed in *Cd4*^−/−^ mice (Cao and DeLeo, 2008). Similar findings were obtained using *Rag2*^−/−^ mice, which did not develop mechanical pain hypersensitivity after SNI surgery. The authors confirmed that *Rag2*^−/−^ mice reconstituted with T cells behave like WT mice in response to SNI (Vicuña et al., 2015). Taken together, these studies indicate a detrimental role for T cells in chronic pain induced by nerve injury. However, there are a few publications showing that SCID and *Rag1*^−/−^ mice developed mechanical allodynia like WT mice in both sexes in response to nerve injury (Sorge et al., 2015; Rosen et al., 2017). A comparison of the infiltration of T cells and the development of pain hypersensitivity after CCI, PSNL, or complete axotomy, found that while all axotomized rats developed pain hypersensitivity, only one third of rats with CCI and PSNL showed allodynia. However, T cells infiltration was observed in the three models and there was no relation between numbers of infiltrating T cells in peripheral nerves and development of allodynia (Cui et al., 2000). In addition, in most publications, the pain hypersensitivity does not correlate with T cell infiltration, as maximal intensity of pain is observed before infiltration and recruitment of T cells. It remains unclear how T-cell-deficient mice are fully protected from SNI (Costigan et al., 2009; Vicuña et al., 2015) while T cells start infiltrating the damaged somatosensory system only several days after injury. These data may suggest an alteration of the early immune response to nerve injury in *Rag1*^−/−^ and *Rag2*^−/−^ mice owing to the impact of T cells on the homeostasis of the innate immune cells.

Treg cells are a particularly interesting subset because they inhibit T cell proliferation and cytokine production. In PSNL-treated mice, injection of anti-CD25 antibody depleted Treg cells in the spleen and lymph nodes and prolonged mechanical pain hypersensitivity (Austin et al., 2012). Targeting CD25 is not specific to the elimination of Treg cells since other immune cells (e.g., monocytes and activated T cells) express CD25 as well. In order to achieve a more effective and specific depletion of Tregs, the same group took advantage of the DEREG mice. DEREG stands for DEpletion of T-REG cells, and in this mouse model, the human diphtheria toxin receptor is expressed under the control of the Foxp3 promoter. When these mice are treated with diphtheria toxin, the Foxp3+ (Treg) cells are specifically depleted. Flow cytometry confirmed Treg depletion, and an increase in CD4+ effector T cells was also observed. Following diphtheria toxin treatment, the DEREG mice showed enhanced mechanical allodynia in response to CCI, with neither the contralateral paw nor the WT mice affected by diphtheria toxin administration (Lees et al., 2015). Thus, Tregs appear to play a protective role in pain after nerve injury.

### Infiltration of T Cells in Diabetic Painful Neuropathy Model

In a model of diabetes type I peripheral neuropathy induced by injection of streptozotocin, T cells infiltrated the DRG at a very late stage. Significant presence of T cells in the DRG was not detected before 19 weeks post-injection, although mechanical pain and spontaneous pain were evidenced earlier (from 8 weeks post-injection; Agarwal et al., 2018). Interestingly, peripheral nerves from patients with diabetic neuropathy showed massive T cell infiltration of the endoneurial and epineurial compartments. In diabetic patients (type I and II) with peripheral neuropathy, approximatively 25 times more CD3+ T cells were counted per section in sural nerve biopsies compared to control patients. The infiltrated T cells were mostly CD8+ T cells and CD25+ cells, an indication of CD4+ or CD8+ Treg (Younger et al., 1996). However, the contribution of T cells to diabetic painful neuropathy has not been investigated yet.

### Contribution of T Cells to Chemotherapy-Induced Peripheral Neuropathy (CIPN)

CIPN is a common side effect of cancer treatment and is often associated with pain. The role of T cells in CIPN has been studied in models of systemic injection of chemotherapeutic agents such as paclitaxel, cisplatin, or oxaliplatin. In a model of paclitaxel-induced neuropathic pain, Liu reported that intrathecal anti-CD8 reduced mechanical allodynia on day 5 and 6 after paclitaxel. This study also showed that intrathecal injection of CD8+ T cells worsened pain hypersensitivity, while injection of Treg cells briefly reduced mechanical allodynia (Liu et al., 2014). These effect might result from the specific route of injection used here, as T cells are not present (or are at a very low level) in the spinal cords of control and CIPN animals (Janes et al., 2015; Denk et al., 2016; Gattlen et al., 2016; Krukowski et al., 2016), in contrast to the experimental autoimmune encephalomyelitis (EAE) model in which a substantial infiltration of T cells is observed in the spinal cord (Rothhammer et al., 2011; Duffy et al., 2019). These beneficial and detrimental effects of Treg and CD8+ T cells, respectively, were not reproduced in transgenic mice. Treg depletion, using the DEREG mice, did not affect pain hypersensitivity after oxaliplatin (Makker et al., 2017). In CIPN induced by either paclitaxel or cisplatin, *Rag1*^−/−^ or *Rag2*^−/−^ female and male mice develop mechanical allodynia with similar intensity to WT mice. Strikingly, the resolution of chemotherapy-induced mechanical allodynia was significantly delayed in the absence of T cells (Krukowski et al., 2016; Laumet et al., 2019). Reconstitution with CD8+, but not CD4+, T cells restored the resolution of mechanical allodynia (Krukowski et al., 2016). While most studies cited above focus only on evoked-pain behaviors, our studies showed that the absence of T cells also impairs the resolution of spontaneous pain assessed by conditioned place preference, and reconstitution with CD8+ T cells normalized the resolution of spontaneous pain (Laumet et al., 2019).

Interestingly, the adoptive transfer of CD8+ T cells into T-cell-deficient mice after CIPN had fully developed failed to promote resolution of pain (Laumet et al., 2019). These findings indicate that the CD8+ T cells have to be exposed to cisplatin in order to be capable of promoting resolution of CIPN. In other words, the CD8+ T cells need to be “*educated*” to acquire the capacity to promote resolution of CIPN by exposure to cisplatin. In support of this idea, adoptive transfer of CD8+ T cells from cisplatin-treated WT mice did indeed promote resolution of established CIPN in *Rag2*^−/−^ mice. This T cell education appears to be independent of antigen recognition by the TCR because reconstitution of *Rag2*^−/−^ mice with CD8+ T cells with mutated TCRs that recognize and respond only to one irrelevant antigen (chicken ovalbumin) retained the capacity to induce CIPN resolution (Laumet et al., 2019). Interestingly, the neuroprotective effects of T cells after brain trauma was also independent of antigen recognition by the TCR (Walsh et al., 2015).

### Contribution of T Cells to Inflammatory Pain

Inflammatory pain is modeled by injection of Complete Freund’s adjuvant (CFA), formalin, or other inflammatory agents into the paw. In response to intraplantar CFA injection, immune cells (CD45+) infiltrate the paw. T cells represented 2%–4% of infiltrated immune cells, and their percentage remain unchanged over in the first 96 h (Rittner et al., 2001) but showed significant increases after 7 days that are maintained for at least 14 days (Ghasemlou et al., 2015). After CFA, the severity of mechanical allodynia was identical in WT and in five different strains of T-cell-deficient mice (nude, *Tcrb*^−/−^, *Tcrd*^−/−^, *Rag1*^−/−^ and *Rag2*^−/−^; Ghasemlou et al., 2015; Sorge et al., 2015; Laumet et al., 2016, 2019; Petrović et al., 2019). These data indicate that inflammatory pain hypersensitivity in the CFA model develops independently of T cells. While the onset and severity of inflammatory allodynia are similar between WT and T-cell-deficient mice, several independent studies reported that the resolution of mechanical allodynia was significantly delayed in T-cell-deficient mice (Boué et al., 2011, 2012; Basso et al., 2016; Laumet et al., 2016). Reconstitution of *Rag1*^−/−^ or *Rag2*^−/−^ mice with WT T cells normalized resolution of CFA-induced mechanical allodynia. Similar findings were obtained after intraplantar injection of IL-1β (Kavelaars lab, unpublished data). The pain behavior in response to formalin was worsened in nude mice compared to WT, and reconstitution of nude mice with CD4+ T cells normalized their response to formalin in both sexes (Rosen et al., 2019). In the antigen- and collagen-induced models of arthritis, CD8+ T cell depletion worsened the pain hypersensitivity (Baddack-Werncke et al., 2017), while in a postsurgical pain model, no alteration in thermal and mechanical hypersensitivity was reported in *Tcrb*^−/−^ and *Tcrd*^−/−^ mice compared to WT mice (Ghasemlou et al., 2015; Petrović et al., 2019). The lack of contribution of γδ T cells to inflammatory pain induced by plantar incision was reported in both sexes (Petrović et al., 2019), and these mice deficient in γδ T cells have a normal pattern of αβ T cells. In conclusion, the existing literature indicates that, in inflammatory pain models, T cells are beneficial or neutral to the pain phenotype.

### Contribution of T Cells to Sex Differences in Pain Signaling

Like most of the preclinical research in pain (Mogil, 2012), the role of T cells has been almost exclusively studied in male rodents, but recent evidence suggests that T cells may contribute to sex differences in pain signaling. Key studies in this area showed that inhibition of microglia relieved nerve injury-induced pain only in male mice (Sorge et al., 2015; Taves et al., 2016; Luo et al., 2018). Critically, this sex difference disappeared in T-cell-deficient mice (*Rag1*^−/−^ and nude mice; Sorge et al., 2015; Mapplebeck et al., 2018). Moreover, a beneficial role of T cells became apparent when comparing pregnant WT and T-cell-deficient mice. In late pregnant WT mice, CFA- and SNI-induced allodynia are suppressed, but this does not happen in T-cell-deficient mice (*Rag1*^−/−^ and nude mice). Adoptive transfer of CD4+ T cells restored pregnancy analgesia (Rosen et al., 2017). T cells are also responsible for the reduced morphine analgesia observed in female mice, and this sex difference in morphine analgesic sensitivity was restored by adoptive transfer of male CD4+ T cells to female nude mice (Rosen et al., 2019). Notably, however, no sex difference was observed in the contribution of CD8+ T cells to CIPN resolution (Laumet et al., 2019). In summary, these data indicate complex interactions between T cells and sex in pain signaling, although the physiology of these interactions remains to be uncovered.

## Targeting T Cells for the Treatment of Chronic Pain

Accumulating literature indicates that T cells contribute to the transition from acute to chronic pain. While in nerve injury models T cells are mostly detrimental, they are mostly beneficial in models of inflammatory pain and CIPN. A potential explanation for this apparent discrepancy may be in the T cell subsets engaged. As mentioned above, Th1 cells are more likely to increase pain, while Th2, Treg, and CD8+ T cells are protective. This would mean that two potential therapeutic strategies can be developed: (i) blocking the pain promoting functions and/or subsets of T cells; and (ii) enhancing the beneficial effects and/or subsets of T cells.

### Potential Mechanisms Underlying the Pain Increasing Effects of T Cells

**Figure 3:**
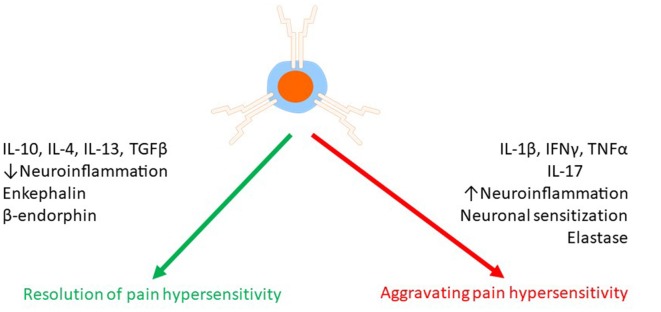
Effects of T cells on chronic pain. T cells can both suppress and promote chronic pain. T cells release a variety of mediators such as pro- and anti-inflammatory cytokines, endogenous opioids, and proteases to regulate pain either *via* a direct effect on pain sensing neurons or indirectly *via* modulation of neuroinflammation.

The pain promoting effect of T cells may result from amplification of neuroinflammation (Figure 3). For example, it has been proposed that Th1 and Th17 cells facilitate macrophage infiltration in the damaged nerve and DRG (Kleinschnitz et al., 2006; Kobayashi et al., 2015). In the spinal cord, *Cd4*^−/−^ mice showed less astrocyte activation at 14 days after SNT (Draleau et al., 2014). In the injured nerve, the infiltrated T cells (Th17 cells) produce IL-17, and this may contribute to microgliosis *via* stimulation of the IL-17 receptors expressed on microglia (Kleinschnitz et al., 2006). Consistent with this model, inhibition of IL-17 signaling reduced microgliosis, mechanical allodynia, and paw flinches associated with bone cancer pain (Huo et al., 2019).

In addition to cytokines, T cells produce the serine protease leukocyte elastase (LE, encoded by the gene *Elane*). LE is released by infiltrated T cells in the DRG after SNI, and it activates matrix metalloprotease 9 (MMP9) which facilitates neuropathic pain (Ferry et al., 1997; Ji et al., 2009). To assess the critical role of LE-producing T cells in neuropathic pain, Vicuña et al. (2015) reconstituted *Rag2*^−/−^ mice with T cells from WT or *Elane*^−/−^ mice and monitored their pain sensitivity following SNI. The lack of LE in the T cells prevented the development of neuropathic pain.

Following nerve injury, infiltrated CD4+ T cells in the dorsal horn of the spinal cord are often associated with increased pain (Cao and DeLeo, 2008; Costigan et al., 2009; Leger et al., 2011). Therefore, a potential therapeutic strategy may be to target the infiltration of the CD4+ T cells into the spinal cord. Repurposing drugs that have been developed to block the infiltration of T cells in the central nervous system in multiple sclerosis may be an attractive strategy to treat neuropathic pain induced by nerve injury. FTY720, a drug used to treat multiple sclerosis, sequesters T cells in the lymph nodes and prevents the infiltration of the nervous system. After PSNL, FTY720-treated mice showed less mechanical and thermal pain sensitivity compared to vehicle-treated mice (Kobayashi et al., 2015). An important caveat is that FTY720 may also reduce pain by mechanisms independent of T cell sequestration (Doyle et al., 2019). Approaches based on blocking α4 integrin to prevent the infiltration of CD4+ T cells into the dorsal horn of the spinal cord are attractive as well (Yednock et al., 1992; Rothhammer et al., 2011), though such antibodies have not yet been tested in chronic pain models. An alternative way to prevent the infiltration of pathogenic CD4+ T cells into the DRG and spinal cord is through surgical sympathectomy (McLachlan and Hu, 2014; Du et al., 2018). Surgical sympathectomy is effective at alleviating neuropathic and inflammatory pain (Agarwal-Kozlowski et al., 2011; Iwase et al., 2012; Xie et al., 2016), but whether this pain relief resulted from blocking T cell infiltration is unknown.

### Mechanisms Underlying the Beneficial Effect of T Cells

Recent studies indicate that T cells also promote the resolution of pain and prevent the transition from acute to chronic pain (Figure 3). The pathways triggered by T cells to resolve pain are not fully understood, but some mechanisms have been elucidated. The subsets of Treg cells, Th2 cells, and suppressor CD8+ T cells have been shown to reduce or resolve pain, and this is likely through their capacity to switch the milieu to an anti-inflammatory environment (Moalem et al., 2004; Austin et al., 2012; Lees et al., 2015; Baddack-Werncke et al., 2017). Importantly, promoting the anti-inflammatory activity of T cells can be achieved by activation of the anti-inflammatory reflex *via* electrical vagus nerve stimulation (Chakravarthy et al., 2015), suggesting a possible translational treatment.

Many neuroprotective and pain resolving effects of CD8+, Th2 and Treg cells could be recapitulated by IL-10 administration and are absent in mice lacking IL-10, pointing to IL-10 as a major player in the beneficial effects of T cells (Frenkel et al., 2005; Liesz et al., 2009; Xie et al., 2015; Krukowski et al., 2016; Laumet et al., 2018; Duffy et al., 2019). IL-10 alleviates inflammation and pain in various chronic pain models (Wagner et al., 1998; Plunkett et al., 2001; Eijkelkamp et al., 2016; Krukowski et al., 2016), and it is possible that T cells act through IL-10 production. However, it is also possible that T cells do not produce IL-10 themselves but induce other cells to synthesize and release IL-10 (Xin et al., 2011; Krukowski et al., 2016). Resolution of mechanical allodynia was similar in *Rag1^−^*^/-^ reconstituted with WT or *Il10*^−/−^ CD8+ T cells, indicating that CD8+ T cells were not the source of the IL-10 required for resolution of pain (Krukowski et al., 2016). Likewise, in models of nerve injury and inflammation-induced depression-like behavior, CD4+ and CD3+ T cells conferred neuroprotection and facilitated resolution by inducing IL-10 production from CNS-resident cells (Xin et al., 2011; Laumet et al., 2018). After spinal cord injury, Th1 cells secrete IFNγ to trigger IL-10 production by macrophages and microglia which will promote resolution of motor deficits (Ishii et al., 2013). Alternatively, in models of acute systemic inflammation, Treg secrete IL-13 to induce IL-10 production by IL-13R+ macrophages (Proto et al., 2018). Thus, how T cells induce the production of IL-10 to resolve pain is not yet understood.

In addition to cytokines, T cells release endogenous opioids to induce analgesia (Kavelaars et al., 1991; Kavelaars and Heijnen, 2000; Sitte et al., 2007; Labuz et al., 2010; Celik et al., 2016; Basso et al., 2018). Endogenous opioids can bind opioid receptors on sensory neurons to dampen pain signaling (Stein et al., 1990, 2003; Labuz et al., 2010). The mRNAs of proenkephalin (encoding the enkephalins) and proopiomelanocortin (encoding the endorphins) can be induced in T cells (Kavelaars et al., 1991; Kavelaars and Heijnen, 2000; Labuz et al., 2010; Boué et al., 2014; Basso et al., 2016). *Ex vivo*, T cells from mice immunized with ovalbumin in CFA produce up to seven time more proenkephalin *Penk* mRNA in response to antigen stimulation than naïve CD4+ T cells (Boué et al., 2011, 2012). *In vivo*, T cells have a critical role in stress-induced analgesia, which is known to be mediated by endogenous opioids. Restraint stress-induced analgesia was absent in athymic nude mice and reduced in WT mice after T cell depletion (Labuz et al., 2006; Rosen et al., 2019). The release of endogenous opioids by T cells during stress-induced analgesia was partly dependent on the receptor for corticotropin-releasing factor (CRF; Labuz et al., 2010). The analgesic effects of T cell-producing endogenous opioids have been investigated in models of chronic pain as well. Infiltrated T cells and other leukocytes in the damaged nerve produce and release opioid peptides (Labuz et al., 2009). Interestingly, while T cells may represent only 11% of infiltrated leukocytes in injured nerves, they constituted approximately 50% of opioid peptide-containing immune cells (Labuz et al., 2010). As mentioned above, pregnancy analgesia (reduced pain sensitivity in the SNI and CFA models in late pregnant mice) was absent in T-cell-deficient mice (*Rag1*^−/−^ and nude) and was restored after adoptive transfer of T cells (Rosen et al., 2017). Rosen et al proposed that T cells promote pregnancy analgesia because they induce upregulation of the *oprd1* expression (δ Opioid Receptor, δOR) in the spinal cord. Indeed, the lack of *oprd1* impaired pregnancy analgesia (Rosen et al., 2017). Similarly, in the CFA model, δOR (but not μOR or κOR) antagonist blocked the endogenous analgesic effect of T cells (Boué et al., 2012). In chronic inflammatory pain models, both CD4+ and CD8+ T cells contribute to endogenous opioid-dependent analgesia and pain resolution (Boué et al., 2011, 2012, 2014; Baddack-Werncke et al., 2017). In contrast to WT T cells, adoptive transfer of T cells from *Penk*^−/−^ mice did not induce resolution of CFA-induced allodynia, suggesting that T cells promote resolution of inflammatory pain by enkephalin release (Basso et al., 2016). Notably, T-cell-derived enkephalins increase the number of Th2 cells and reduced the numbers of Th1 and Th17 cells (Boué et al., 2014; Basso et al., 2018). These findings indicate that in addition to their direct analgesic effects, endogenous opioids released by T cells may also suppress pain *via* their anti-inflammatory effects.

**Figure 4:**
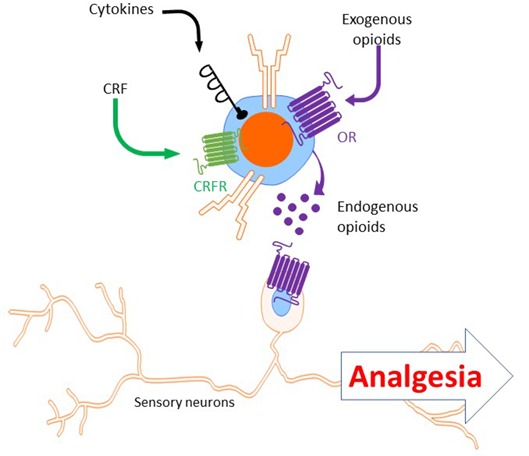
Analgesia induced by T cells secreting endogenous opioids. Upon stimulation with CRF, cytokines or exogenous opioids, T cells release endogenous opioids (e.g.: enkephalins, β-endorphin). Endogenous opioids released by T cells bind opioid receptors (e.g.: μ- and δ-opioid receptors) on sensory neurons to induce analgesia. CRF, corticotropin releasing factor; OR, opioid receptors.

Beside the role of T cells in endogenous analgesia, T cells play a role in pain relief induced by exogenous opioids. T-cell-deficient mice (*Rag1*^−/−^, nude and *Cd4*^−/−^ mice) showed reduced morphine analgesia in the formalin and tail-withdrawal tests. Reconstitution with CD4+ but not CD8+ T cells restored morphine analgesia (Rosen et al., 2019). T cell-mediated endogenous analgesia is stimulated by administration of exogenous opioids, as T cells increase the production and release of endogenous opioids in response to exogenous opioid (Labuz et al., 2006; Boué et al., 2012; Celik et al., 2016; Figure 4). Finally, administration of synthetic opioid agonists in the damaged nerve produces analgesia which is dependent of infiltrated leukocytes [as mentioned above, 50% of opioid-producing leukocytes are T cells (Labuz et al., 2010)].

### Reprogramming T Cells Toward a Pro-resolution Phenotype

There are a variety of potential pathways to promote a pro-resolution phenotype in T cells, many of which are sensitive to existing compounds. T cell subsets are not stable and can be “re-fated” upon appropriate stimulation. For example, Th17 cells naturally acquire an anti-inflammatory phenotype to then become IL-10-producing Tregs to resolve inflammation in various models of chronic inflammation (Gagliani et al., 2015). This plasticity presents an attractive therapeutic opportunity to switch pain promoting Th1 and Th17 cells to a phenotype that promotes resolution of pain and inflammation such as Treg or Th2 cells.

Glatiramer acetate (GA), a drug with good safety profiles and tolerability used to reduce the frequency of multiple sclerosis relapse, has immunomodulatory properties (Dhib-Jalbut, 2003; Arnon and Aharoni, 2004; Blanchette and Neuhaus, 2008). GA increased the number of IL-10-producing CD4+ T cells in the dorsal horn of spinal cord, reduced the activation of microglia, and alleviated allodynia in models of inflammatory and neuropathic pain (Sharma et al., 2008; Leger et al., 2011).

Experimentally, Treg response can be amplified by treatment with the superagonist of the B7 receptor for co-stimulation: CD28 (supCD28). In the CCI model, supCD28 administration expanded the number of Treg cells in the injured sciatic nerve and spinal cord. SupCD28-stimulated Tregs reduced the number of macrophages in the sciatic nerve and the DRG and decreased astrocyte and microglia activation in the spinal cord as well. SupCD28 did not affect the onset of CCI-induced mechanical allodynia but accelerated its resolution (Austin et al., 2012).

Another way to stimulate the pro-resolution T cell pathway could be *via* vaccination with CNS-restricted self-antigens (Schwartz and Moalem, 2001). After axotomy, immunization with myelin-derived peptide (myelin oligodendrocyte glycoprotein: MOG) stimulated neuron survival by recruiting autoreactive T cells to the site of injury (Moalem et al., 1999; Hauben et al., 2000a,b). The beneficial effects MOG immunization may rely on IL-10 producing CD4+ T cells (Frenkel et al., 2005). However, despite the high incidence of chronic neuropathic pain after nerve injury, the immunization strategy has not yet been tested in chronic pain models.

**Figure 5:**
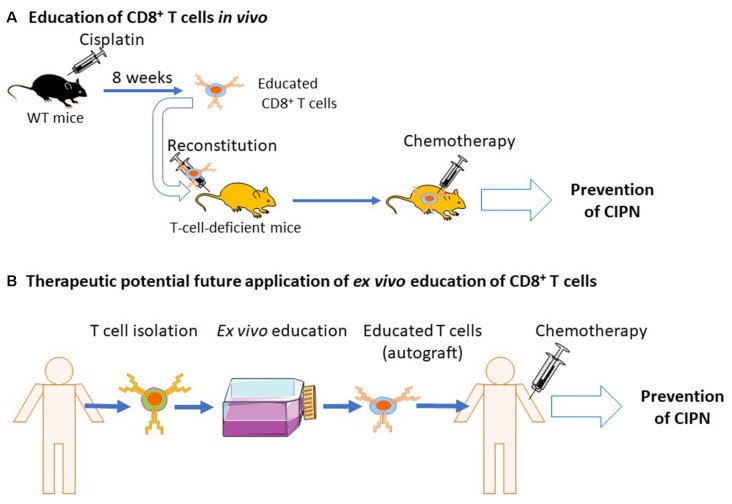
Education of CD8+ T cells by cisplatin and potential clinical translation. **(A)** Naïve mice are treated with cisplatin and allowed to recover from chemotherapy-induced peripheral neuropathy (CIPN). Now educated, CD8+ T cells are isolated and injected into T-cell-deficient mice. The recipient mice, reconstituted with educated CD8+ T cells, are now protected from CIPN (Laumet et al., 2019). **(B)** Potential future clinical applications of educated CD8+ T cells. T cells are collected from cancer patients before chemotherapy. It may be possible to educate T cells in *ex vivo* cultures to acquire a pro-resolution phenotype. Educated CD8+ T cells could then be re-injected to the same patient as an autograft which may protect the patient from CIPN.

CD8+ T cells are mostly beneficial in animal models of inflammatory pain and CIPN (Krukowski et al., 2016; Baddack-Werncke et al., 2017). As described above, in order to resolve CIPN, CD8+ T cells need to be educated. Interestingly, adoptive transfer of educated CD8+ T cells before chemotherapy prevented the development of pain in response to cisplatin or paclitaxel treatment (Laumet et al., 2019). If we can develop ways to educate CD8+ T cells *in vitro* to promote resolution of pain, one could envision that CD8+ T cells from a patient with CIPN can be educated *ex vivo* to acquire a pro-resolution phenotype and be re-injected as an autograft to the same patient to treat CIPN (Figure 5).

## Future Directions

The significant growth of our knowledge of the involvement of T cells in the transition from acute to chronic pain in the last few years highlights the complexity of its disparate beneficial and pain aggravating effects. In order to make further progress in our comprehension of the role of T cells in chronic pain, it is necessary to investigate other Th subsets (e.g., Th9 and Th22) and identify phenotypic profiles of T cells in patients suffering from chronic pain and CIPN as well as in animal models. These T cell profiles may be diverse, with specific features for different chronic pain conditions. Thus, identifying a T cell signature of chronic pain could inform the search for treatment targets for specific groups of patients. Alternatively, a recent study measured DNA methylation in circulating T cells at 9 months after peripheral nerve injury. The authors showed genome-wide changes in DNA methylation in circulating T cells. Intriguingly, these changes in the T cells methylome remarkably overlapped (72%) with the DNA methylation modifications in the prefrontal cortex (Massart et al., 2016). Nerve injury reprograms DNA methylation in the peripheral and central nervous systems, and these changes in DNA methylation are linked with pain hypersensitivity and comorbid depression-like behavior (Tajerian et al., 2013; Garriga et al., 2018). Thus, assessing epigenetic changes in circulating T cells may provide a non-invasive window to uncover epigenetic modifications in the peripheral and central nervous systems associated with chronic pain.

In addition to identifying potential biomarkers, targeting T cells offers the potential to develop disease-modifying therapy. The development of T cell-based therapy would have the potential to not only dampen neuroinflammation but also promote repair and permanent recovery from chronic pain. An important issue for the development of T cell-based therapy for chronic pain is the recognition of antigens by the TCR. Whether T cells need to recognize an antigen for their beneficial or detrimental effects on pain is an open question. We demonstrated that CD8+ T cells do not need to recognize a specific antigen to induce resolution of CIPN (Laumet et al., 2019). In contrast, T cells do need to recognize an antigen to facilitate the release endogenous opioid to alleviate inflammatory pain (Boué et al., 2011, 2012). The potential requirement of antigen recognition for resolution of pain would influence how we could engineer T cells to treat chronic pain. Additionally, signaling molecules (e.g., chemokines) that recruit T cells and their cellular source are of great interest as well, as they represent another attractive therapeutic target. Pharmacological modulation of chemokine signaling may allow us to selectively attract pro-resolution T cells to the site of injury and block the infiltration of pathological pain promoting T cells.

It is interesting to point out that T cells also contribute to the resolution of depression-like and anxiety-like behaviors (Cohen et al., 2006; Lewitus et al., 2009; Brachman et al., 2015; Clark et al., 2016; Laumet et al., 2018), two disorders that are frequently co-morbid with chronic pain. Thus, a dysfunctional T cell-mediated endogenous resolution system may be the link between chronic pain and its psychiatric comorbidities, and a thorough understanding of the role of T cells may help resolve not only chronic pain, but also comorbid mental disorders.

## Author Contributions

GL drafted the manuscript. GL and AK designed the review. JM, AR, SK, CH and AK provided critical inputs.

## Conflict of Interest

The authors declare that the research was conducted in the absence of any commercial or financial relationships that could be construed as a potential conflict of interest.
